# Machine learning techniques for lipid nanoparticle formulation

**DOI:** 10.1186/s40580-025-00502-4

**Published:** 2025-07-15

**Authors:** Hao Li, Yayi Zhao, Chenjie Xu

**Affiliations:** 1 Department of Biomedical Engineering, College of Biomedicine, City University of Hong Kong, Tat Chee Ave, Kowloon, Hong Kong SAR China; 2 Institute of Digital Medicine, City University of Hong Kong, Tat Chee Ave, Kowloon, Hong Kong SAR China

**Keywords:** Lipid nanoparticle formulation, Ionizable lipids design, Machine learning, Nucleic acid therapy

## Abstract

A significant amount of effort has been poured into optimizing the delivery system that is demanded by novel therapeutic modalities. Lipid nanoparticle presents as a solution to transfect cells safely and efficiently with nucleic acid-based therapeutics. Among the components that make up the lipid nanoparticle, ionizable lipids are crucial for the transfection efficiency. Traditionally, the design of ionizable lipids relies on literature search and personal experience. With advancements in computer science, we argue that the use of machine learning can accelerate the design of ionizable lipids systematically. Assuming researchers in lipid nanoparticle synthesis may come from various backgrounds, an entry-level guide is needed to outline and summarize the general workflow of incorporating machine learning for those unfamiliar with it. We hope this can jumpstart the use of machine learning in their projects.

**Graphical Abstract:**

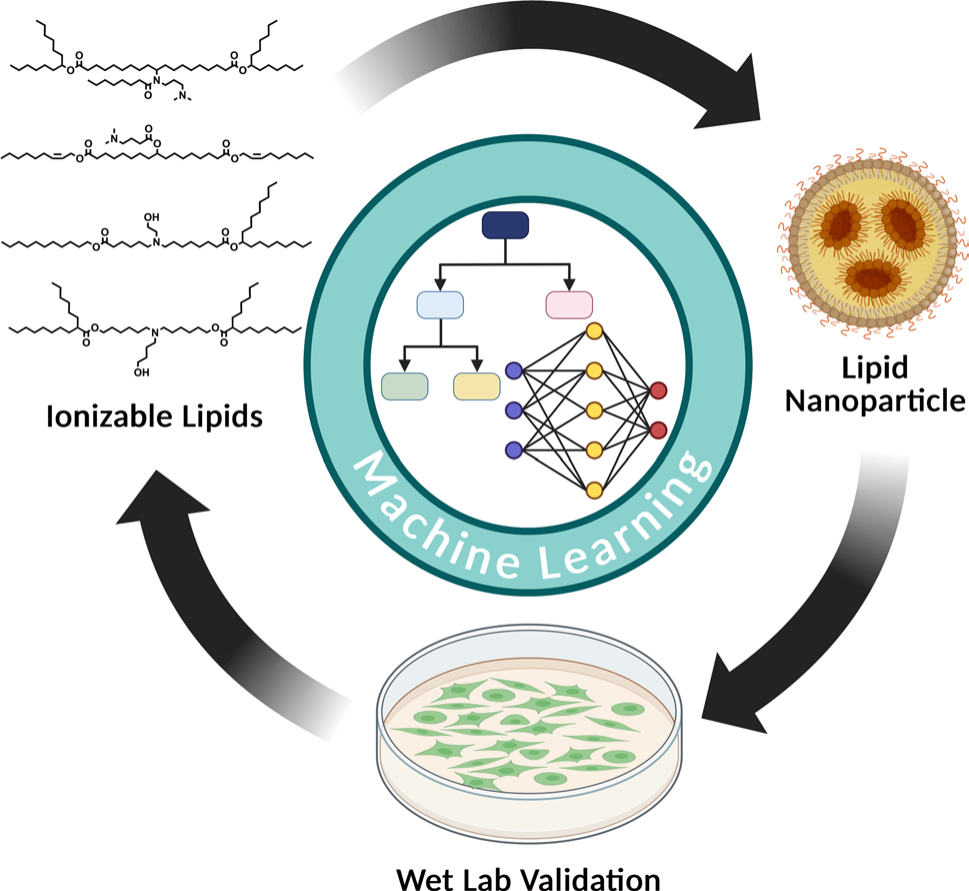

## Introduction

Gene therapy promises a paradigm shift in medicine, and the two most important attributes of gene therapy are safety and efficacy in the delivery of nucleic acid payloads into target cells [1, 2]. Many delivery systems are thus developed to fulfil this dual mandate by improving the stability and circulation time of drugs in vivo and facilitating their cellular entrance. These delivery systems can be generally grouped into two categories: viral vectors and non-viral approaches. Viral vectors such as lentiviruses and adeno-associated viruses (AAVs) are highly efficient in transfection but pose safety concerns. Historically, serious adverse effects—such as leukemia—have been reported in retrovirus-based gene therapy patients [3, 4]. AAVs, on the other hand, are considered to be rarely carcinogenic and serve as the platform for most FDA-approved gene therapeutics [5]. Notwithstanding the safety of AAVs, their small packaging capacity of ~ 4.5 kilobases limits the donor size [6]. Non-viral approaches include physical methods such as electroporation and chemical methods such as nanoparticle-based carriers. They are advantageous in term of relative safety, ability to transfer large size genes, and easiness for preparation, among others. However, they are limited by low transfection efficiency and poor transgene expression. To improve transfection efficiency, efforts have been made to develop new gene carriers and techniques. One of the star performers is lipid nanoparticle (LNP), highlighted by the success in delivering COVID-19 mRNA vaccines (Comirnaty and Spikevax) and the first siRNA drug (Patisiran) [7–9].

LNPs are typically made up of four essential components: ionizable lipids for endosomal escape and nucleic acid protection; helper phospholipids for the stable lipid bilayer formation, with the most common being distearoylphosphatidylcholine (DSPC); cholesterol to maintain the rigidity and fluidity of LNPs; polyethylene glycol (PEG)-lipids to prevent premature removal from the body by the immune system [10]. Despite their great importance, helper phospholipids, cholesterol, and PEG-lipids have well-defined lipid structure/composition, making their main tunable attribute their concentrations. Conversely, the chemical structure of ionizable lipids is far more versatile than the aforementioned three and therefore being widely explored [11]. Ionizable lipids vary in lipid chain length, branching, headgroup type, and molar ratios, leading to tens of thousands of potential formulations [12, 13]. These formulations of ionizable lipids contribute to various attributes of the resulting LNPs, among which we consider the safety and functionality more important than others [2]. From a safety perspective, it is essential to ensure that LNPs made from ionizable lipids exhibit good biocompatibility, low immunogenicity, and minimal toxicity to the liver and other organs [2]. From a functional standpoint, it is necessary not only to achieve efficient delivery of mRNA, siRNA, and other nucleic acids in vitro, but also to ensure adequate cellular uptake and endosomal escape in vivo in specific organs—such as the lungs, spleen, bone marrow, tumors, heart, and the brain—made possible with tailored ionizable lipids [2, 14–16].

Given the prolific attributes of LNPs derived from ionizable lipids, the structural versatility of ionizable lipids highlights a major challenge they present: the burden of choice—how do we determine the best ionizable lipids to use? Traditional approaches rely on the comparison of different ionizable lipids through manual repetitive synthesis that is time-consuming and labor-intensive. This eventually would resort to senior lab members’ “Just do it” and “It works best”. The stark shift of artificial intelligence landscape is providing the potential to supercharge ionizable lipids and thus LNP design through rapid selection and examination of ionizable lipid compositions from existing knowledge as well as derivatives from machine learning (ML). Oftentimes, the experimental researchers working with LNP synthesis may not be familiar with how they can implement ML in their workflow. While there are reviews on using ML for LNP studies, we find their breadths and depths not well-aligned with the needs of entry-level researchers, making them difficult to read. To save time and effort for these audiences, we create this guide that introduces ways of implementing ML to accelerate ionizable lipid discovery, as demonstrated in recent primary research articles, while also providing a summary of the algorithms. As we limit the scope of this guide to ionizable lipid, we can then clearly indicate the steps readers should take and recommend specific software and platforms for readers to play with. This clear structure aims to make the concept of using ML more accessible to first-time users.

**Fig. 1:**
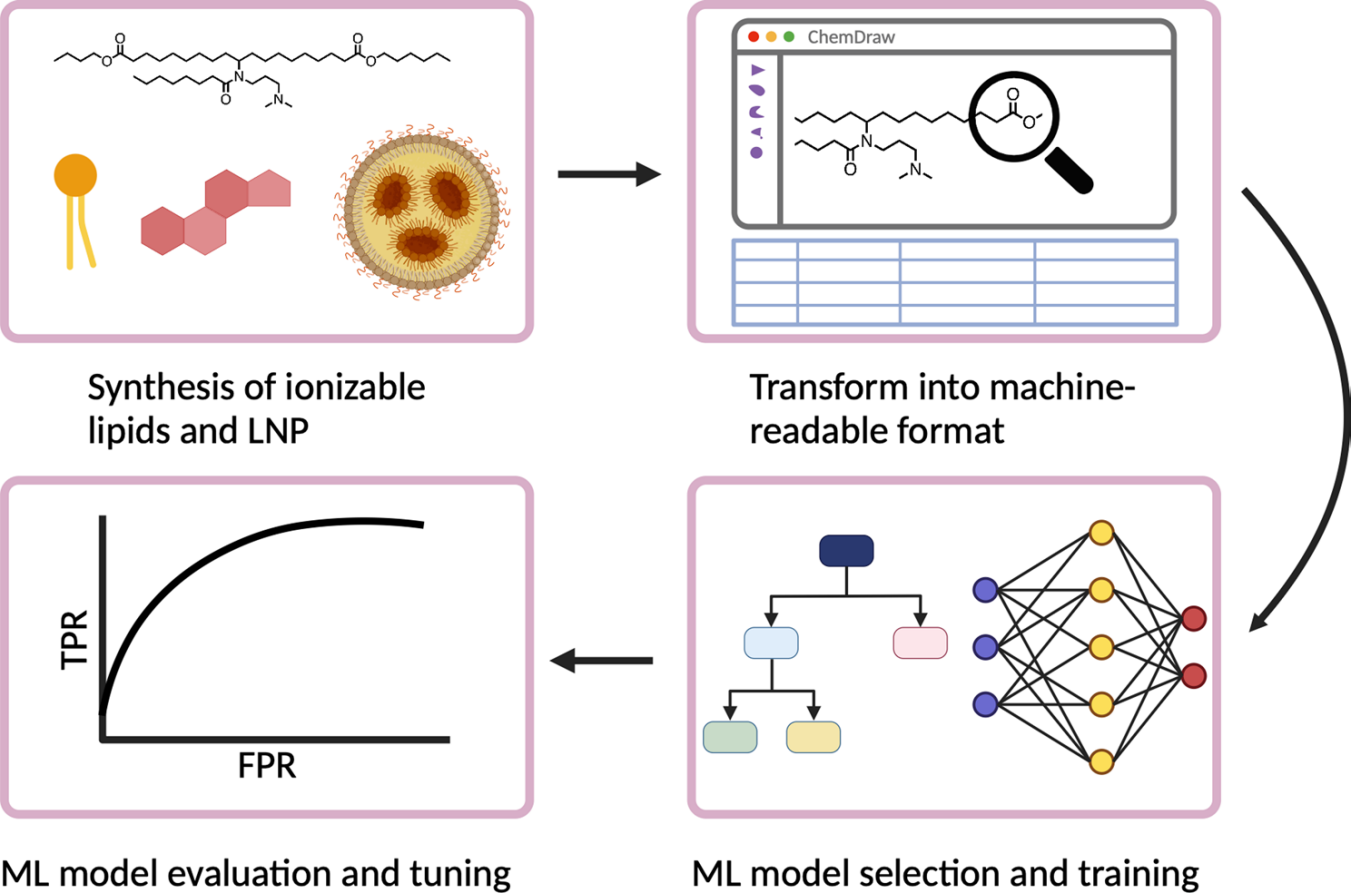
Overall workflow of utilizing ML for ionizable lipid and LNP development. Generated using BioRender

To begin, we regard the complete cycle of ML for ionizable lipid and LNP design consisting of the following four steps: synthesis of ionizable lipids and LNPs, transformation of data into machine-readable formats, ML model selection and training, and evaluation and refining of the ML models (Fig. 1).

## Data collection: synthesis of ionizable lipids and LNPs

Given the endless possibilities of their compositions, identifying and manufacturing ionizable lipids with good safety and transfection efficiency is one of the very first challenges in LNP design. We know that in order to train a ML model, massive amount of experimental data is required. To achieve this, quick and efficient synthesis would expedite the discovery and validation of new ionizable lipids. Several groups have explored simplified ways to streamline the production of different ionizable lipids with slight modifications. For example, there are traditional methods such as addition of acrylates or acrylamides to primary or secondary amines, as well as epoxide ring opening with amines [17–20]. These strategies, while quick and easy, are short of flexibility in sidechain arrangement as they are made with the addition of mere two components [19, 21]. In 2019, researchers explored the Ugi reaction to couple amines, isocyanides, and alkyl ketones in a one-pot high-throughput synthesis of ionizable lipids [21]. Later, the same group developed a one-step four-component reaction that enables increased dimensionality and improved yields, which makes the synthesis of more varieties of ionizable lipids a possibility [19]. In both cases, Ugi reaction resulted in bis-amide formation which may be more stable and less biodegradable than ester bonds [22]. Using Passerini reaction, Xu et al. developed a linker with hydrolysable ester group on the top of the possible ester groups in side chains [23]. Having additional ester may increase biodegradation and shorten circulation time, thus improving the biosafety of ionizable lipids [22]. However, ester groups closer to the header-linker region can significantly decrease therapeutic potency and negatively impact shelf-life [24, 25]. These syntheses are facile and catalyst-free; yet some groups pointed out that adding alkyne in ionizable lipids can boost transfection efficiency and opted for a one-pot synthesis with metal catalyst required for alkyne reduction, namely A^3^-coupling [26–28]. Regardless, these advances in high-throughput synthesis provide solid footing to generate a wide array of different ionizable lipids for ML algorithms.

To further save cost, apart from the high-throughput experiments, provisional ionizable lipid library can be plenished virtually. Using open-source cheminformatics platforms such as RDKit, KNIME, and DataWarrior, researchers can apply SMIRKS or SMARTS-encoded reaction rules to automate the generation of chemically valid and synthetically accessible virtual lipid structures, thereby significantly expanding the ionizable lipids library [29]. For example, Xu et al. designed virtual lipid libraries using combinatorial chemistry approaches such as the Ugi three-component reaction (3-CR), enabling the systematic variation of head groups, linkers, and alkyl chains [30]. Virtual libraries created *via* the Markush editor in ChemAxon Marvin Suite can be exported as SMILES strings and subsequently converted into molecular graphs using RDKit, allowing the topological features of the lipid molecules to be effectively captured [30]. Reaction-based enumeration and optimization can then be performed in KNIME using RDKit nodes, such as the “RDKit One Component Reaction” node, to generate new lipid candidates. The generated libraries can be further refined through rule-based filtering and molecular descriptor calculation, and ultimately, ML or deep learning models can be applied to predict the delivery efficiency of LNP formulations containing the candidate lipids [30].

In terms of assembling the four components, namely ionizable lipids, helper phospholipids, cholesterol, and PEG-lipids into LNPs, the current best practice is microfluidics, which is favored for its precision, scalability, ease of use, and consistent yield [19, 22, 31–33]. Besides microfluidics, there are also chemical and physical methods for synthesizing LNPs (Table 1). Chemical methods include solvent evaporation, film hydration, and dialysis [34]. However, they are limited by the complexity of fabrication, difficulty in controlling particle size and polydispersity, poor reproducibility between batches, and the risk of solvent residue contamination [35–37]. Physical methods include sonication, extrusion, and centrifugation, suffering from limited control over the nanoscale structure and potential damage to sensitive biomolecules like mRNA [38, 39]. Yet, in microfluidic devices, biomolecules are subjected to much lower shear stresses than in sonication and extrusion methods, and this low mechanical stress maintains the structural integrity of sensitive nucleic acids, which are susceptible to degradation under harsh conditions [40]. Also, microfluidic system rapidly and uniformly mixes organic phase and aqueous phase using accurate control of fluid speed within microchannels, allowing for precise control over nanoparticle size, distribution, and surface properties. Microfluidic technology also supports scalable and continuous production, ensuring stability and reproducibility for large-scale manufacturing [41, 42]. With this stability, batch-to-batch variation of LNPs is reduced, thus reducing noise in algorithm training, and improving predictive performance.

**Table 1:** Summary of LNP synthesis methods

Method	Description	Advantages	Disadvantages
Microfluidic Mixing	Combines lipid and aqueous phases in microchannels for controlled nanoparticle formation [43, 44]	Precise control of particle size & PDI; Excellent reproducibility & easy scaleup; Minimal solvent residue [45]	High capital cost of equipment; Prone to channel clogging; Batch size limited by microchannel dimensions [46]
Solvent Evaporation	Lipids dissolved in organic solvent are evaporated to form a thin film, then hydrated with aqueous mRNA solution [47]	Well-established, simple setup; Can produce large batches per run [48]	Broad size distribution; Risk of residual organic solvent; Relatively slow and labor‑intensive [49]
Film Hydration	Similar to solvent evaporation; involves drying lipid film and rehydrating with mRNA solution [50]	No specialized instrumentation required; Gentle, room‑temperature process [50]	Poor size control; Lower encapsulation efficiency; Manual, laborintensive [51]
Dialysis	Lipid and mRNA solutions are mixed and dialyzed to remove solvents, facilitating nanoparticle formation [52]	Very mild—minimal mRNA damage; Efficient removal of smallmolecule reagents [53]	Extremely slow—hours to days; Hard to scale up; Poor control over particle selfassembly kinetics [54]
Sonication	Uses ultrasonic energy to disperse lipids and form nanoparticles [55]	Widely accessible equipment; Quick reduction of aggregate size [55]	High energy can shear/damage mRNA; Broad size distribution; Low reproducibility [56]
Extrusion	Forces lipid-mRNA mixture through membranes with defined pore sizes to control particle size [57]	Very uniform particle size; Able to easily tune mean diameter through membrane pore size [55]	Requires multiple passes/timeconsuming; Membrane fouling/clogging; Shear stress may degrade mRNA [56]
Centrifugation	Applies centrifugal force to separate nanoparticles based on size and density [55]	Simultaneous concentration & purification; Efficient removal of both large aggregates and small debris [58]	Not truly a synthesis step - mainly for purification; Long spin times; High‑speed spin can induce aggregation or damage [58]

After synthesis, the LNP’s physical and chemical characteristics must be examined. The particle size and polydispersity index (PDI) are studied with dynamic light scattering (DLS) by analyzing the fluctuations in scattered light caused by Brownian motion. This evaluates the colloidal stability and homogeneity of LNP formulations [59]. The morphology of LNPs is imaged using cryogenic transmission electron microscopy (Cryo-TEM) by preserving the native hydrated state of the sample through rapid vitrification, allowing for detailed profile of particle size, shape, lamellar organization, and encapsulation of active pharmaceutical ingredients [60, 61]. Nuclear magnetic resonance (NMR) spectroscopy can be used to examines the behavior of LNPs in solution and the chemical structures of lipids and lipid-nucleic acid interactions. For example, ^1^H NMR enables the quantification of PEG density and conformation on the surface of LNPs [62, 63].

Besides LNP’s physical and chemical properties, we should screen their functional performance through a defined set of criteria. Encapsulation efficiency, representing the proportion of nucleic acids in the particles, is usually expected to exceed 80%. The apparent pKa of the ionizable lipid, is ideally between 6.0 and 7.0, as it plays a central role in facilitating endosomal escape *via* pH-sensitive protonation [64, 65]. Then biological performance of LNPs would be evaluated through in vitro transfection on model cells. For example, Zhu et al. established a multi‑step screening platform that systematically evaluates various helper lipids and component ratios, enabling the identification of liver‑targeted, highly efficient, and long‑acting plasmid DNA LNP formulations from over 1,000 candidates, and further extends their transgene expression in mouse liver *via* a DNA/siRNA co‑delivery strategy [66]. Similarly, Xu et al. synthesized LNPs loaded with firefly luciferase mRNA with 1200 different ionizable lipids, and quantified the transfection potential [30]. The transfection potential data was then used to train the AGILE ML model and establish its relationship with molecular properties of LNPs [30].

## Data transformation

Following the synthesis and data collection of ionizable lipids and LNPs, we need transform the chemical information in the structure of a molecule into machine readable format for feeding the ML algorithms. This can be achieved through programs capable of calculating molecular descriptors that represent the properties of a given chemical structure. Molecular descriptors are calculated or estimated using statistics, chemometrics, and chemoinformatics [67]. These values can be inputted into an algorithm to develop quantitative structure-activity relationship (QSAR) models, which can be used to infer, for example, the transfection efficiency of LNPs.

The first step is to draw the structure of synthesized ionizable lipids. This not only documents lipids, but also produces a machine-readable chemical file. There is a range of softwares available for this, such as ChemDraw, ChemSketch, and MarvinSketch. All of these allow for the generation of chemical files like MDL molfile that has the X-Y-Z coordinates of atoms, or SMILES which only offers connectivity information.

The chemical files are sent to softwares that calculate molecular descriptors. Two of the most commonly used tools are PaDEL-descriptor and RDKit [12, 19, 30, 68, 69]. PaDEL-descriptor is an open-source software designed to compute a comprehensive set of 797 1-D, 2-D, and 3-D descriptors, as well as 10 types of fingerprints, using The Chemistry Development Kit [69]. Thanks to its graphical user interface and straight-forward descriptor output, it is very easy to use; it is also suitable for large-scale descriptor calculations credited to its multithreaded design that enhances performance [69]. RDKit only offers command-line interface, requiring users to have basic Python skills and to look through their documentations [70]. However, RDKit has much more functions and integrations built-in, making it a more versatile tool than PaDEL-descriptor. For example, apart from calculating descriptors, researchers can use RDKit to transform text-based chemical files directly to molecular graphs without additional software like ChemDraw, then input to the neural network with ease [30]. Despite their differences, both offer open-source access with comprehensive documentations to assist users. Additionally, detailed guidance is provided for customizing the calculation of pre-set or self-defined descriptors, should users require them for enhanced machine learning training.

Furthermore, the graphical representation of molecules can be utilized alongside molecular descriptors to enhance training optimization. Alternatively, it can serve as a standalone input for algorithms [12, 30, 71]. This requires graphs to be transformed to machine-readable fingerprints using graph neural network (GNN), which will be introduced in the next section. These fingerprints, like molecular descriptors, can then be used as inputs in ML algorithms.

## ML model

### Model selection and training

After we screened the performance of LNPs and transformed each component in the LNP to values, the next step is to train ML algorithms. The connections between the descriptors and attribute-of-interest can be deciphered using several algorithms. The simplest and the most interpretable model is linear regression (Table 2), which identifies a linear relationship between the attribute-of-interests and LNP properties. Its simplicity and interpretability come at the cost of predictive accuracy [72]. Researchers have also developed several non-linear models commonly used to improve predictive accuracy: support vector machine, random forest, gradient tree boosting like XGBoost, and various neural networks [12, 19, 72, 73]. These models use pre-labelled input and output to be trained to make predictions using regression (for continuous numerical values) and classification (for discrete class values).

**Fig. 2:**
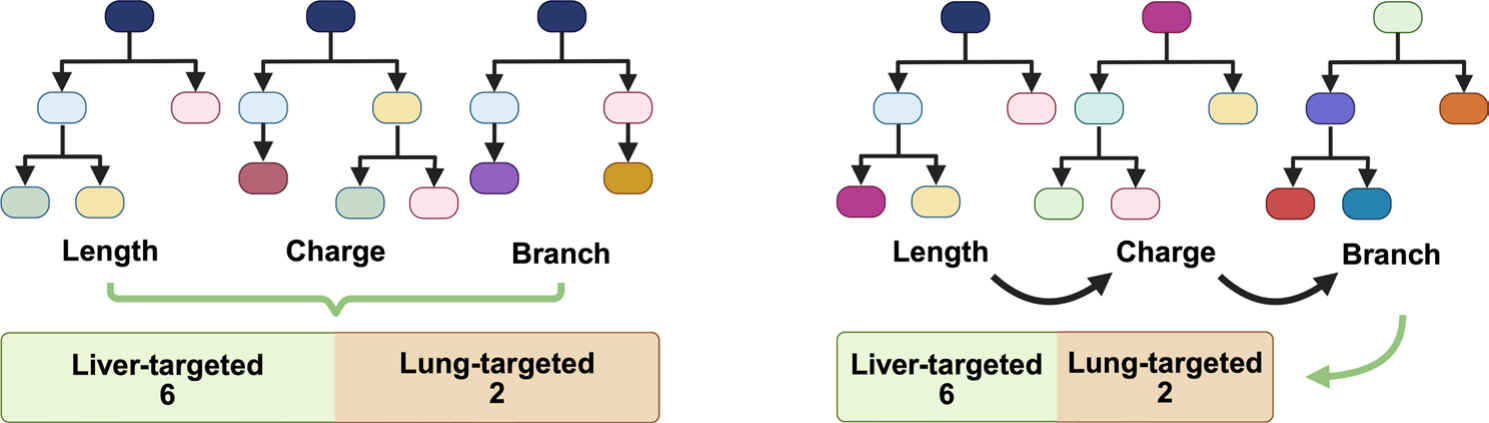
Toy example of parallel tree (left) and sequential tree (right) models. Generated using BioRender

In general, these non-linear models (Table 2) can be categorized as either tree-based, instance-based, or deep learning [72]. A tree-based algorithm connects input data to the outcome through a series of decisions, like a flowchart. Each decision splits data into smaller groups based on the difference in value or classification; through recursive splitting, a tree-like structure called decision tree forms, leading to the final prediction. An example would be random forest, where multiple trees are generated at the same time (parallel trees) using randomly selected subsets of data (Fig. 2). The aggregate outcome of all trees would then be used to decide, using majority voting for classification, or averaging for regression. On the other hand, gradient boosting like XGBoost instead generates decision trees sequentially with each additional tree corrects the error of the previous tree, improving accuracy [73].

**Fig. 3:**
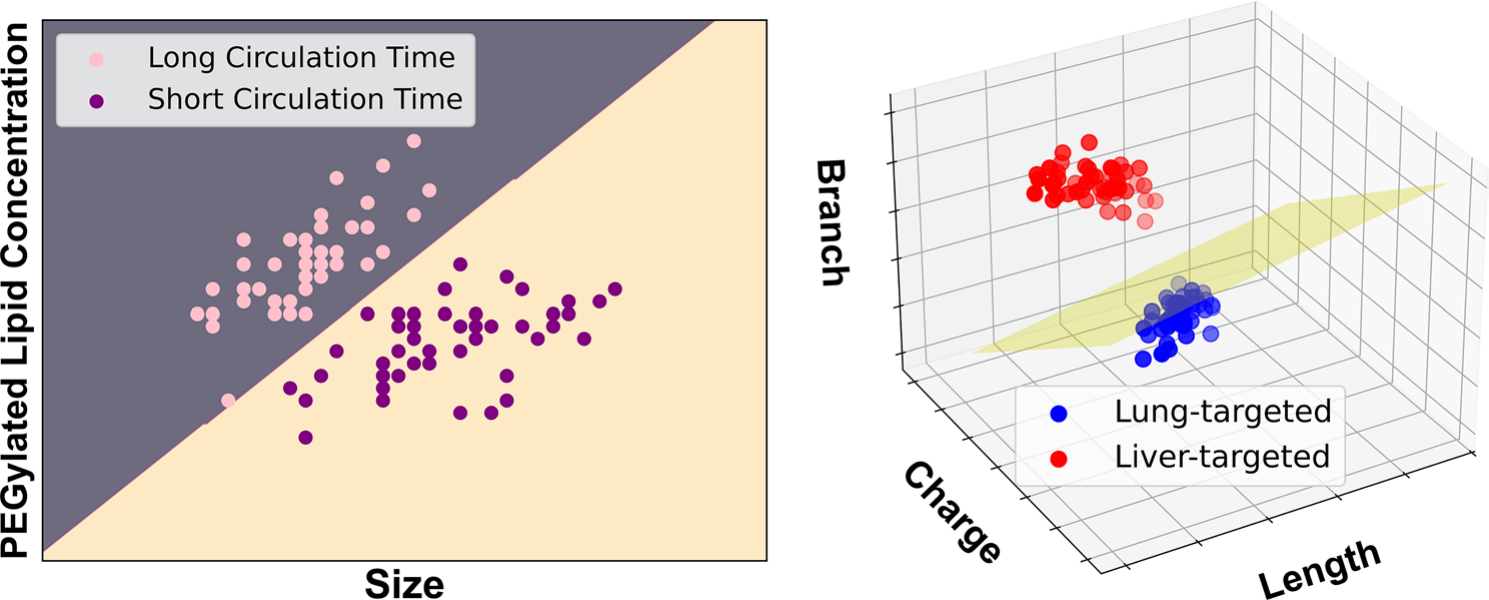
Toy example showing linear SVM decision boundaries in 2-D (left) and 3-D (right). Generated with sample dataset in scikit-learn [75]

An instance-based algorithm classifies new data points based on their similarity to existing data points. An example would be the support vector machine (SVM). It can transform complex 2-D data, where finding a linear boundary is difficult, into 3-D or higher-dimensional space where data become separable by a hyperplane, making classification easier [74]. In a toy example below (Fig. 3), 2-D plot would separate the data into 2 classes even though there was no clear boundary for these two classes, meaning there might be error in the classification. Yet, once a third attribute was added that increases the dimension of data, a hyperplane (as the plane is more than 2-D) would be able to segregate data into “Lung-targeted” and “Liver-targeted” quite distinctively.

**Fig. 4:**
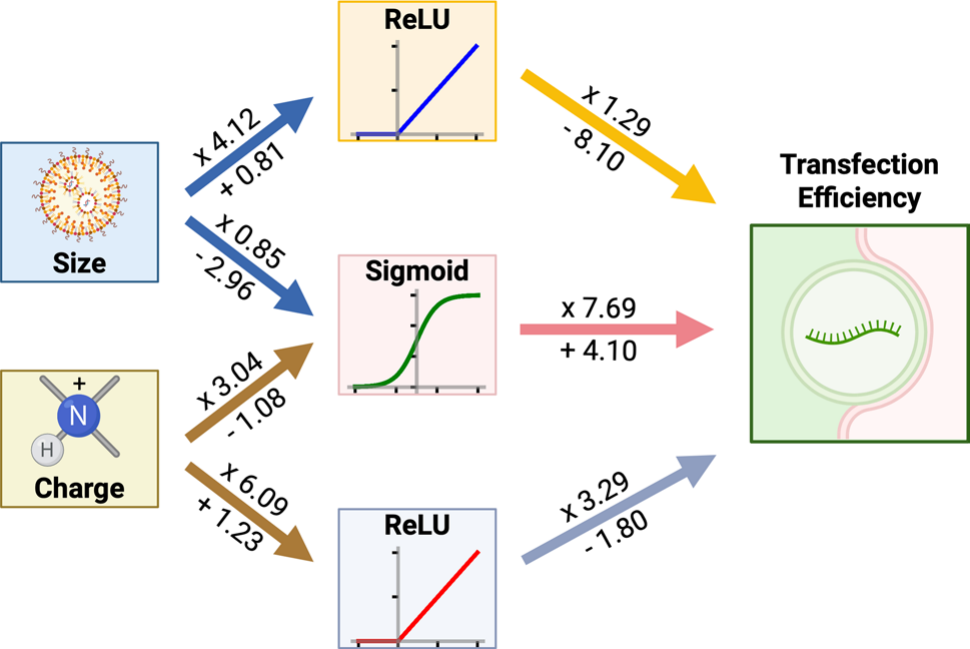
A toy multi-layer perceptron model consisting of 3 layers, from the input features of size and charge, to the output of transfection efficiency. Features are connected to nodes with activation functions, either ReLU or sigmoid, with weights and biases applied. Generated using BioRender

A deep learning algorithm uses neural networks. A neural network, such as multi-layer perceptron (MLP), uses neurons and weights to make predictions (Fig. 4). Neurons would get weighted inputs from the previous neurons, and then apply these inputs to a function. Such function is usually ReLU, softmax, or sigmoid to introduce non-linearity. A collection of neurons can form highly complex curves that no one function can describe, improving a neural network’s ability to fit data and make accurate predictions. This collection of neurons forms a layer within the network. Neurons would then send the computation result to next layers of neurons. With more layers, the process becomes much less interpretable while the accuracy on complex tasks increases.

**Table 2:** Summary of machine learning models

ML Technique		Description	Key traits	Interpretability
**Linear Regression**		A simple linear model used for binary classification	Simple, fast, assumes linear relationships	Intrinsic; entirely interpretable [76]
**Non-linear regression**				
Instance-based	Support Vector Machine (SVM)	An instance-based model that finds the classification hyperplane	Effective in high-dimensional spaces, kernel trick for non-linearity [74]	Post-hoc; requires interpretability methods [76]
Tree-based	Random Forest	Parallel decision trees generated using randomly selected data	Reduces overfitting, robust, handles categorical and numerical data [77]	Intrinsic; requires interpretability methods with large trees [76]
	Gradient Tree Boosting (e.g. XGBoost)	Sequential decision trees generated to minimize error	Highly accurate, handles missing data well [73]	Intrinsic; requires interpretability methods with large trees [76]
Deep learning	Multi-layer perceptron (MLP)	A feedforward neural network with non-linear activation functions at neural nodes	Supervised learning through backpropagation that captures complex patterns [78]	Post-hoc; requires interpretability methods [76]
	Graph Neural Networks (GNN)	A group of neural networks designed to process graph-like data	Works with irregular, graph-like data [79]	Post-hoc; requires interpretability methods [76]
	Convoluted Neural Network (CNN)	A GNN specialized in dealing with grid-like data	Spatial hierarchies, translation invariance, widely used in vision tasks [80]	Post-hoc; requires interpretability methods [76]
	Graph Transformer (e.g. Grover)	A model capable of self-supervised training on graphs	Captures long-range dependencies in graphs, powerful for complex structures [81]	Post-hoc; requires interpretability methods [76]

In the previous section, we described ways of transforming molecular structures into machine-readable, text-based descriptors to be inputted to the ML model of selection. Nonetheless, we foreshadow that graphs, instead of text-based descriptors, can be used directly with GNN to implement molecular representation learning. For GNN, a graph $\:G=(V,E)$ describes a connection between a set of nodes ($\:V$) and a set of edges ($\:E$), which for a molecular representation would be a set of atoms (nodes) and a set of bonds (edges) [12, 71]. The earliest graph neural network model would be the famous convolutional neural network (CNN) that extract spatial features using convolution filters on grids. While handling grid-like structures incredibly well, it cannot handle edges with different lengths, for example, a molecule. Later development of spectral graph convolution uses graph Fourier transform enabling convolution on graphs rather than mere grids; However, the complexity of using Fourier transform limits the range of possible applications [80]. Graph convolutional network reduced the computational cost and storage cost to scale linearly in the number of graph edges instead of quadratically; yet it does not use edge features [82]. To handle both node and edge features, message passing neural network was developed [83]. Latest GNN model for chemistry and biology includes Grover, which is pre-trained on a large collection of molecules with self-supervised pre-training, enabling it to learn general representations and make predictions on molecule properties [12, 81].

### Hardware and skill consideration

Comparing across the descriptor- and graph-based models, researchers have reported better interpretability and computational efficiency on descriptor-based models and better performance when trained on smaller datasets for graph-based models; oftentimes the predictions yielded from different models are drastically different with no significant differences in terms of accuracy [12, 71]. It should be noted that each model has different time complexity (i.e. how much time is needed with regard to increasing data). For example, graph transformer has a complexity of $\:O\left({n}^{2}\right)$ where 𝑛 is the number of nodes per graph (e.g. atoms in a molecular graph). This means the time needed to train scales quadratically with increasing nodes. To use this in combination with, for example, non-linear SVM, where the complexity is in the range of $\:O\left({n}^{2}\right)$ to $\:O\left({n}^{3}\right)$ ($\:n$ = number of samples), can take a significant amount of time with a large dataset. Below we summarized a table (Table 3) with rough system requirements and time costs for various models based on 1000 lipid samples with 3000 attributes for each sample. Time cost estimates are based on previous experimental results. Notwithstanding the report showing improved training outcome by the combined used of descriptor- and graph-based models, researchers should decide which approach—or the combination of—best fits their workflow and resource/time constraints without compromising model performance [30].

**Table 3:** Summary of system requirement and time of machine learning models [84]

Model	Model Type	Data Type	CPU	Discrete GPU	RAM	Approx. Training Time	Approx. Tuning Time
SVM	Instance-based	Tabular	Multi-core (8 + cores)	Optional	8 + GB	Seconds to minutes per epoch	Minutes
Random Forest	Tree-based	Tabular	Multi-core (8 + cores)	Optional	8 + GB	Seconds per epoch	Minutes
XGBoost	Tree-based	Tabular	Multi-core (8 + cores)	Optional	16 + GB	Seconds per epoch	Minutes
MLP	Deep learning	Tabular	Multi-core (8 + cores)	CUDA GPU recommended	16 + GB	Minutes per epoch	Hours
GNN	Deep learning	Graph	Multi-core (8 + cores)	CUDA GPU with high memory recommended	16 + GB	Minutes to tens of minutes per epoch	Hours
Graph Trans-former	Deep learning	Graph	Multi-core (16 + cores)	CUDA GPU with high memory recommended	32 + GB	Minutes to hours per epoch	Hours

Implementing these algorithms requires basic coding skills in various languages. Some algorithms, such as random forest and XGBoost, have easy-to-use packages on R and Python. Users only need to tweak some parameters to perfect the training process. Neural networks require more changes in hyperparameter tuning thus can be harder to deploy; and sometimes it would be hard to determine how changing the hyperparameter can help the results. To help researchers build their own neural networks, many packages—such as TensorFlow and scikit-learn—have been developed. Although reading documentation and coding sound daunting, with the comprehensive documentation and the vast number of tutorials online, researchers with no coding background can use template codes to start playing with basic ML models.

### Data splitting

Before proceeding to training the model, a portion of the data yielded should be set aside for validation. Considering we are training the model on a limited dataset to minimize the time and effort required for data generation, there is a risk that the model may fit the training data too precisely, leading to reduced accuracy when applied to real-world data. This is an overfit, which we describe in the next section. It is debatable regarding to how much data is to be split between the training and validation set; A researcher argues that it is intuitively associated with how many parameters there are in a regression model and should be $\:\sqrt{p}\::1$ in a linear regression, where $\:p$ is the number of parameters [85]. Most researchers that implemented ML in LNP reported a three-way split of training, validation, and test data at the ratio of 8:1:1 [12, 15, 30, 68].

## Tuning and evaluating the model

**Fig. 5:**
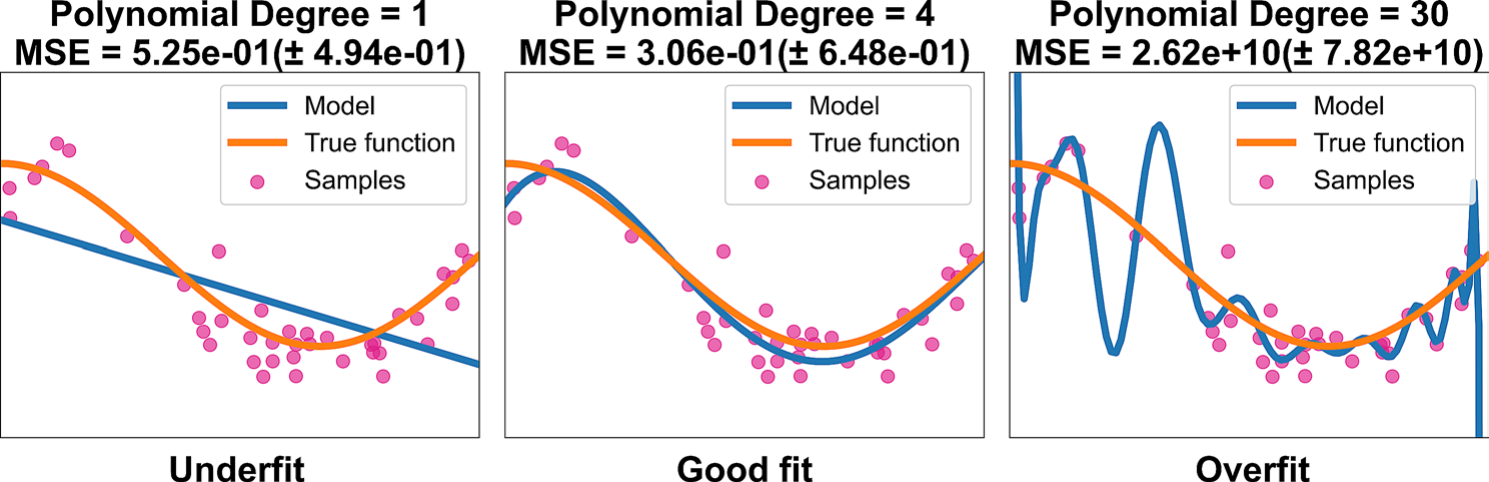
A graph showing that underfit and overfit can happen when using polynomial regression to approximate nonlinear functions. These models have different degrees of polynomial features, with a linear function having the degree of 1. The higher the mean squared error, or MSE as depicted on the graph, the less likely the train model would generalize to actual data [75]

Using the test split of data that we set aside, we judge the performance of models on several grounds: its accuracy, precision, and recall in classification tasks; its error, bias and variance, in regression tasks; and overall the cost of running [86, 87]. By analyzing these metrics, we can spot potential weaknesses in the models, such as biased predictions, high variance, or poor recall in certain cases. For regression algorithms, bias and variance are important attributes because they indicate how well the model learns from training data and generalizes to new data. Low variance and high bias models oversimplify the data, causing underfitting (Fig. 5), which prevents the model from identifying important patterns and produces subpar prediction performance. On the other hand, overfitting describes the case where variance is high and bias is low (Fig. 5). In this instance, the model does not generalize to other datasets because it learns the training data too well, including the noise and small variations. Even though the accuracy on training set may be quite good, it can still perform poorly on test and real data.

To strike a balance between bias and variance, techniques such as regularization and hyperparameter tuning are needed. Hyperparameters, like the learning rate in SVM and neural network, can be tuned using grid search or random search to find the best combination that minimize error. Regularization also helps solving the overfitting issue by essentially “desensitizing” the model which neglects unhelpful parameters, noise, and outliers. Simple techniques are used such as dropout, where neurons in neural networks will be randomly dropped thus removing some functions and weights that define the model, resulting in a thinned network that is more generalized [88, 89]. Early stopping is another very direct method in which a validation set would be used to identify an inflection point where the error instead increases with more iteration; The last registered parameters before such point would be selected as the optimal model parameter, instead of further iterations that will lead to more errors due to overfitting [88]. By iteratively refining the model and evaluating its performance on an additional validation set reserved during data splitting, we can develop models that not only fit the training data well but also maintain high accuracy when generalizing to test sets and real-world data.

Apart from a test set, a standardized benchmark can be used to examine the performance on different sets of molecules. The test sets used by researchers are often made in-house or different in each experiment. Doing testing on these set when the algorithm is train on the same population warrants a good outcome; however, not running a standardized benchmark would be the bane of other researchers when it comes to comparing new algorithms with those previously reported, since different datasets, their pre-training preparation like splitting methods, and evaluation metrics cannot be compared directly [12, 90]. The lack of benchmarks makes it difficult to determine whether a performance improvement is from genuine algorithmic advancements or simply from the variation in dataset selection and preparation. To address this problem, one of the benchmarks designed to compare the performance of algorithms for chemistry- and physiology-centric tasks is MoleculeNet [90]. It provides a collection of curated datasets with well-defined evaluation protocols, thus enabling a consistent comparison between different machine learning models. Given the diverse span of MoleculeNet’s datasets across physical chemistry, physiology, and quantum mechanics, it allows researchers to assess their lipid nanoparticles prediction models comprehensively. Apart from MoleculeNet, researchers can turn to widely used ionizable lipids benchmarks such as MC3, ALC, and SM102 used in Moderna’s Spikevax that have their attributes well-documented to test for the performance of their models [19, 30, 91]. Moreover, more researchers are pushing towards publicly disclosing new datasets with transfections efficiency, among other attributes, providing the community with high-quality data to be trained or tested on [12, 15, 30]. In conclusion, such benchmarks allow for comparison across different data reporting thus displaying if the improvements are truly reflective of algorithmic progress rather than dataset-specific optimizations.

When evaluating the model, we remind that improved interpretability greatly benefits not only the researchers who are tuning the models, but also readers of publications produced from these models in understanding which variable or treatment is important to changing the outcome. This is especially true when the expected benefits from using the model are not entirely result-oriented, but to gain insights. Most non-linear models explained herein (Table 2), such as high-dimensional SVM, large decision trees, and deep learning neural networks, do not have good interpretability. Their complexity, reliance on numerous parameters, and non-linear decision boundaries make it challenging to understand how individual features contribute to the predictions. This lack of transparency poses challenges in scientific research, regulatory environments, and clinical applications where interpretability is crucial for trust and reproducibility. To address this issue, an open source book “Interpretable Machine Learning: A Guide for Making Black Box Models Explainable” well-explained the strategies available to researchers to make these “black box” models more transparent, ranging from feature attribution methods like Shapley additive explanations (SHAP) and local interpretable model-agnostic explanations (LIME) to the use of surrogate models, which approximate the behavior of complex models using simpler, more interpretable ones [76, 92, 93]. These approaches help researchers better visualize complex decision-making processes, allowing for better model tuning and more explicit communication of findings to other readers, fostering broader acceptance and applicability of ML in fields where explainability is paramount.

**Fig. 6:**
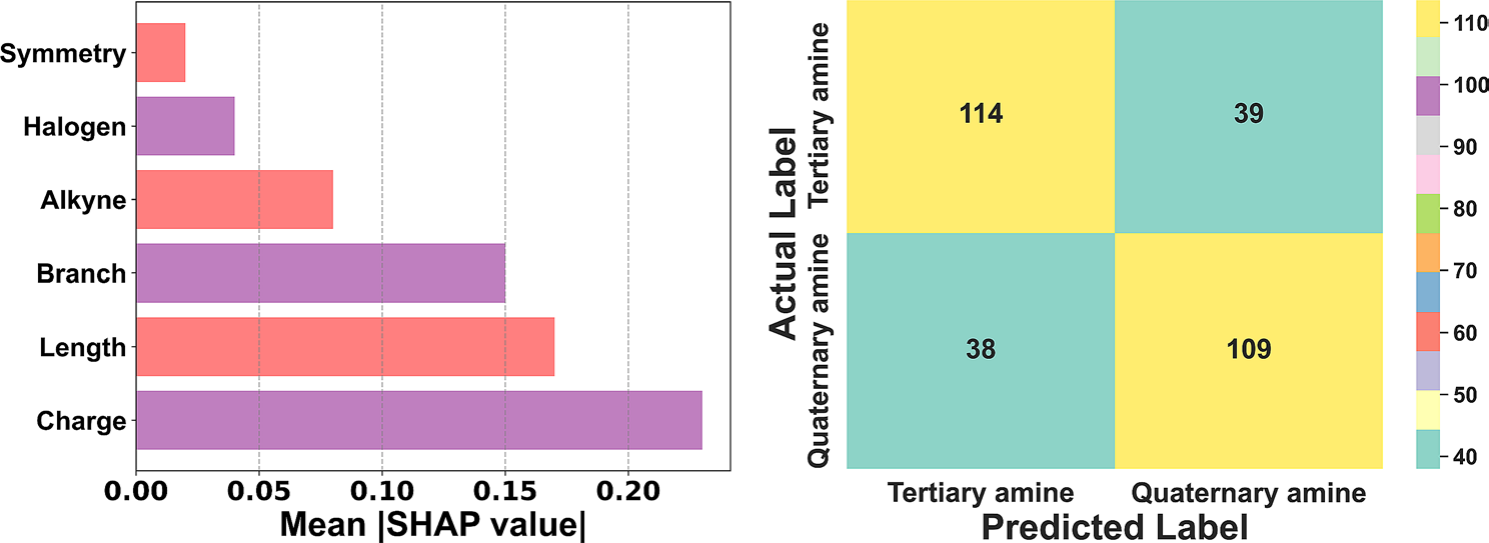
Increased interpretability through visualization of average absolute SHAP values (left) indicating how impactful a feature is on a model, and improved understanding of classification accuracy (right) using a toy random forest model [76]

Of course, following iterations of tuning and evaluating, we must dedicate some man labor to validate predictions in web lab. It is crucial to ensuring the computational predictions match well to the actual biological outcomes. These LNPs must be manufacturable, then characterized for their physical traits such as size and p*K*_a_. Then in vitro validation can be testing the predicted transfection efficiency, efficacy, and cell specificity in controlled conditions. This can involve techniques such as flow cytometry and quantitative PCR to measure gene expression levels post-delivery. In vivo validation to profile the success of gene editing or transient protein level modification would be done with immunoassays. These validations enable the researchers to scope into the model’s predictive capabilities and provide feedback for further refining the computational approach.

## Conclusion

This short review highlighted key principles for using ML in the design and synthesis of ionizable lipids and, eventually, LNPs. ML is transforming scientific research, and its potential in LNP development is huge—but for researchers without much ML experience, getting started can feel overwhelming. Our goal with this simple review is to make the process more approachable, helping researchers plan their experiments with confidence.

As mentioned earlier, while other reviews exist, they often present ML in ways that are too technical or unfocused for newcomers. We believe, to truly harness the power of machine learning in lipid nanoparticle research, we must make a guide understandable and reachable to a broader audience of scientists. This review is meant for that, to serve an educational purpose: to help lower the barrier of entry for researchers who are curious about ML but lack formal training. By narrowing our scope to ionizable lipids and presenting concrete examples, recommended platforms, and a clear workflow, we hope that we have clarified the process and provide some practical guidance. Broadcasting ML knowledge to more researchers—especially those in experimental fields—will not only democratize its usage but also foster interdisciplinary collaborations that can drive innovation.

Of course, we recognize that ML comes with its limitations. One of the biggest issues with any ML model is data scarcity. In LNP studies, this scarcity stems from the lack of good publicly available labelled data, as well as the limited amount of data one single lab could possibly produce due to the strenuous wet lab synthesis [94]. Data scarcity has a range of negative impacts on ML: overfitting, poor generalization, and unreliable predictions… These challenges manifest in ionizable lipid design as such: overfitting may cause predicted ionizable lipids to be overly structurally similar to top performing examples in training set; poor generalization could limit predicted ionizable lipids to be only suitable for a specific cargo like mRNAs, or only suitable for delivering to a specific organ instead of another; unreliable prediction may result in the predicted ionizable lipid candidates failing wet lab validation. Given the nature LNP studies, we can mitigate the issue by using a hybrid method that uses the descriptor-based model to supplement the graph-based models, or vice versa, as discussed previously [30, 95]. We can also be more careful in choosing ML models: since data need increases drastically with more complex ML model such as deep learning, simpler ML models like SVM, random forest, and gradient tree boosting would be more appropriate for a small data scenario; a review article by Xu et al. nicely summarizes the steps to take when working with smaller data sizes [96]. But most importantly, we call on researchers to make labelled data publicly available, enabling others to enrich their data pool.

Therefore, we want to acknowledge and appreciate the researchers who have made their code and data public. Their contributions make it easier for those new to ML to build their own models without starting from scratch [12, 15, 30, 97]. Sample models like AGILE and LiON provide a strong foundation that allow researchers to tweak and evolve their approaches rather than recoding a model from the ground up [15, 30]. As ML continues to gain traction in LNP research, we encourage researchers in the field to explore how these tools can enhance their work. Integrating ML can streamline processes, improve predictions, and even lead to unexpected discoveries. Just as importantly, sharing results and methodologies with the wider community will help push the field forward faster. By working together and embracing new technologies, we can accelerate progress in lipid nanoparticle research and beyond.

## Data Availability

Toy data set used in the Fig. 3 can be retrieved on https://github.com/scikit-learn/scikit-learn/blob/98ed9dc73a86f5f11781a0e21f24c8f47979ec67/sklearn/datasets/data/iris.csv.

## Abbreviations

AAV: Adeno-associated virus
FDA: United States Food and Drug Administration
LNP: Lipid nanoparticle
mRNA: Messenger ribonucleic acid
siRNA: Small interfering ribonucleic acid
DSPC: Distearoylphosphatidylcholine
PEG: Polyethylene glycol
ML: Machine learning
TPR: True positive rate
FPR: False positive rate
PDI: Polydispersity index
DLS: dynamic light scattering
Cryo-TEM: Cryogenic transmission electron microscopy
NMR: Nuclear magnetic resonance
DNA: Deoxyribonucleic acid
QSAR: Quantitative structure-activity relationship
GNN: Graph neural network
SVM: Support vector machine
MLP: Multi-layer perceptron
ReLU: Rectified linear unit
CNN: Convoluted neural network
CPU: Central processing unit
GPU: Graphics processing unit
RAM: Random-access memory
SHAP: Shapley additive explanations
LIME: Local interpretable model-agnostic explanations
PCR: Polymerase chain reaction
